# The relationship between neurocognition and symptomatology in people with schizophrenia: social cognition as the mediator

**DOI:** 10.1186/1471-244X-14-138

**Published:** 2014-05-13

**Authors:** Bess YH Lam, Adrian Raine, Tatia MC Lee

**Affiliations:** 1 Laboratory of Neuropsychology, The Jockey Club Tower, The University of Hong Kong, Rm656, Pokfulam Road, Hong Kong, China; 2 Laboratory of Cognitive Affective Neuroscience, The University of Hong Kong, Hong Kong, China; 3 Departments of Criminology, Psychiatry, and Psychology, University of Pennsylvania, 3809 Walnut Street, Jerry Lee Centre of Criminology, Philadelphia, PA 19104, USA; 4 The State Key Laboratory of Brain and Cognitive Sciences, The University of Hong Kong, Hong Kong, China; 5 Institute of Clinical Neuropsychology, The University of Hong Kong, Hong Kong, China

**Keywords:** Cognition, Faux pas, Eyes test, Theory of mind, Schizophrenia, Emotion

## Abstract

**Background:**

The relationship between neurocognition and symptomatology in people with schizophrenia has been established. The present study examined whether social cognition could mediate this relationship.

**Methods:**

There were 119 participants (58 people with paranoid schizophrenia and 61 healthy controls) participated in this study. Neurocognition was assessed by Raven’s Progressive Matrices Test, the Judgment of Line Orientation Test, and the Tower of London Test. Psychiatric symptoms in people with schizophrenia were assessed by the Positive and Negative Syndrome Scale. Social cognition was measured by the Faux Pas Test, the “Reading the Mind in the Eyes” Test, and the Interpersonal Reactivity Index.

**Results:**

Results were consistent with previous findings that neurocognition and social cognition were impaired in the clinical participants. A novel observation is that social cognition significantly mediated the relationship between neurocognition and symptomatology.

**Conclusions:**

These findings suggest that neurocognitive deficits predispose people with schizophrenia to worse psychiatric symptoms through the impairment of social cognition. Findings of the present study provide important insight into a functional model of schizophrenia that could guide the development of cost-effective interventions for people with schizophrenia.

## Background

Neurocognitive impairment is a well-studied feature of schizophrenia [1-4], and its association with functional impairment has been well validated [4-8]. For instance, people with schizophrenia scored lower on executive functioning, which is one aspect of neurocognitive functioning when compared to the healthy controls. Also, this impairment was related to negative symptoms in these people [4]. However, prior studies often collapsed and treated neurocognition as one single domain [9]. Since neurocognition is a broad construct encompassing visual and verbal learning, executive functioning, processing speed, and memory, further studies on the relationship between this construct and functional impairment or symptomatology are needed. The association of neurocognition with negative symptoms has been widely studied [4,10,11], while positive and general symptoms have received much less attention in prior studies. The meta-analysis in 73 studies revealed that negative symptoms were associated with worse neurocognition and functional outcomes in schizophrenia [11]. Specifically, negative symptoms were related to deficits in problem solving, reasoning, executive functioning [4,12,13], and IQ [14]. However, the findings regarding the association between neurocognition and positive symptoms were scarce and inconsistent [11,15,16]. According to Ventura and colleagues [11], positive symptoms were not significantly related to neurocognition and functional outcomes [17], while Rabinowitz and colleagues found the opposite [18]. Taken together, these findings suggest that neurocognition is more closely associated with negative than with positive symptoms. Moreover, general symptoms were often neglected in prior studies. Therefore, further studies are warranted for the investigation of the relation of neurocognition to symptomatology, including negative, positive, and general symptoms in schizophrenia. Prior longitudinal studies found that neurocognitive deficits predicted the onset of psychosis [19,20] as well as negative symptoms at the 1-year follow up in first-episode psychosis [14]. Specifically, the review [19] has revealed that neurocognitive deficits exist prior to the onset of psychosis. These findings were consistent with the argument by Comblatt and Keilp [21] that neurocognitive deficits lead to social and emotional impairment, and this is reflected in the negative symptoms in schizophrenia. Hence, we hypothesized that neurocognitive deficits predicted the severity of symptoms instead of the other way around in the present study [11].

Because neurocognition alone could account for only 20–60% of the variance in functional outcomes [7], social cognition, which is a common correlate of both constructs [6], has been speculated to be a potential candidate to explain the neurocognition-functional outcomes relation [9,22]. Social cognition, which is also impaired in people with schizophrenia [23,24], covers a range of constructs: theory of mind (ToM) or self-representation, emotion perception or empathy, and social perception [25]. Specifically, regarding ToM, people with schizophrenia performed worse than healthy controls in the Faux Past Test after controlling for age and gender [26] as well as in the “Reading the Mind in the Eyes” Test [27]. In terms of empathy as measured by Interpersonal Reactivity Index (IRI), these people scored worse in two subscales (perspective taking and personal distress), but there was no significant difference in the other two subscales (fantasy and empathetic concern) [28]. These suggest that some aspects of empathy might be retained even in neurocognitively impaired individuals.

A review of 15 prior studies showed that various social-cognitive domains, including ToM, emotion perception, empathy, and social perception, were significant mediators in such a relation [9]. Also, [29-31] found that social cognition assessed by objective measures such as the Facial Emotion Identification Test [32] and the Half-profile of Nonverbal Sensitivity [33] played a significant role in the functional outcomes in schizophrenia as well as in the clinical high-risk-for-psychosis group [34]. These findings suggest that neurocognitive impairment may have an adverse impact on social-cognitive functioning, which in turn predisposes people with schizophrenia to poor functional outcomes. More importantly, it leads to the speculation that social cognition may also mediate the relation between neurocognition and symptomatology in schizophrenia [35]. However, this hypothesis was scarcely examined.

Moreover, prior studies regarding social cognition suffered from a common limitation: they often focused on only one domain of the multi-dimensional social cognition construct at a time [9]. It was found that only 3 out of 15 studies investigated more than one domain of social cognition [9]. Among these studies, relatively few were done to explore empathy, which is also considered as one domain of social cognition [36], and the findings have been inconsistent. For instance, empathy assessed by IRI, which is a self-report scale, was found to be negatively associated with negative symptoms in schizophrenia in some studies [37,38]. However, its association with symptomatology encompassing negative, positive, and disorganized symptoms was not significant in other studies using the same assessment, IRI [39,40]. These inconsistent results might be due to two factors: (1) Different types of psychotic disorders were examined in these studies. Specifically, people with schizophrenia [37,38] and people with first-episode psychosis [39] were examined respectively. (2) Self- ratings of social cognition might be more biased when compared to the objective measures [29,31] in schizophrenia. Further studies are warranted to address these inconsistencies and speculations.

The present study extended previous studies by examining a broader concept of social cognition and symptomatology and separating neurocognition into various factors. Prior studies have focused on certain neurocognitive aspects such as executive functions, working memory, and attention and its relation with symptomatology in schizophrenia, while other aspects such as visual and spatial learning and memory were overlooked [11]. Therefore, the present study investigated perception, which has been less investigated in prior literature. Besides that, executive functioning, which is the core neurocognitive impairment in schizophrenia and shares substantial variances with working-memory capacity (*r* = .97), was also examined [41]. All in all, the primary goals of the present study were to examine the neurocognitive (executive functioning and perception) and social cognitive (theory of mind and empathy) abilities and to examine whether social cognition played a mediating role in the relation of neurocognition to negative, positive, and general psychopathological symptoms in schizophrenia. It was hypothesized that both of these abilities were impaired in schizophrenia but not in healthy controls. Moreover, it was hypothesized that theory of mind as well as empathy would mediate the association between neurocognitive abilities (executive functioning or perception) and symptomatology (negative, positive, and general symptoms) in schizophrenia.

## Methods

### Participants

One hundred and nineteen Chinese participants (58 people with the diagnosis of paranoid schizophrenia and 61 healthy controls) with a mean age of 40.62 years (SD = 9.23 years) were recruited. The inclusion criteria for healthy controls (male = 31, female = 30) from the community were the following: (1) no history of mental health illness, head injury, neurological disorders, or medical diseases that might have affected brain functioning; (2) no history of substance or alcohol abuse, or of mental deficiency; and (3) normal or corrected-to-normal vision and hearing. The paranoid schizophrenia group (male = 29, female = 29) was recruited based on the following criteria: (1) either inpatients or outpatients of a local psychiatric hospital; (2) diagnosed with paranoid schizophrenia with the Diagnostic and Statistical Manual of Mental Disorders, Fourth Edition (Text Revision) [42]; (3) no history of head injury, neurological disorders, or medical diseases that might have affected brain functioning other than paranoid schizophrenia; (4) on antipsychotic medications; and 5) not diagnosed with other co-morbid Axis I or II conditions.

Age, education, and sex were matched across the two groups. The mean age for healthy controls and the people with schizophrenia were 41.3 years (SD = 9.5 years) and 40.0 years (SD = 9.0 years), respectively. The healthy controls attained a mean of 11.3 years of education (SD = 2.7 years) while the schizophrenia group had a mean of 10.4 years of education (SD = 3.0 years). The schizophrenia group with the mean duration of illness of 13.4 years (SD = 8.8 years) and mean onset age of 25.9 years (7.5 years) included both inpatients (*n* = 23, 39.7%) and outpatients (*n* = 35, 60.3%). These two subgroups did not differ significantly in terms of age, duration of illness, age of onset, and education, but did differ in the proportion of sex (inpatients: 16 male and 7 female; outpatients: 13 male and 22 female). The ethics approval of the current study was granted by the New Territories West Cluster Clinical Research and Ethics Committee. Upon the receipt of a written informed consent from the participants, they were assessed with the following measures (except that symptomatology was only assessed in the schizophrenia group).

### Measures

The Tower of London Test (TOL) [43] was administered to assess executive functioning, particularly with regard to planning, processing, and problem-solving skills. In this task, a set of three beads was first strategically placed on three rods with descending heights, and participants were then asked to rearrange the beads from a preset starting position using a different set of beads and rods to match the original set of beads. Participants’ performances were measured in terms of the accuracy of the solution (TOL accuracy), the efficiency of the solution (TOL time), and rule breaks during the solution (TOL score). The accuracy was measured by the rate of correct moves while the efficiency was indexed by the total time taken to solve all solutions. This measure was reported to be sensitive to problem difficulty [44]. The less time used, the more efficient the participant was. the TOL score was calculated by how compliant the participants were with the three basic rules: (1) do not put more than the number of balls allowed on any peg, (2) only place the balls on the peg but nowhere else, and (3) only move one ball at a time.

Judgment of Line Orientation Test (JLOT) [45] contains 30 items that assess visuo-spatial processing. In this test, the participants were presented with stimuli, each consisting of 11 lines separated by an angle of 18°. They were then asked to match a pair of lines with the array of 11 stimulus lines. Each correct answer was given one point, and the total score was thereby computed.

Raven’s Progressive Matrices Test (RPM) [46], which is designed for assessing general intelligence, was administered to all participants. RPM has been widely used in practice and research, and it consists of a series of diagrams or designs with a part missing. Participants were given a number of choices, which were placed below the diagrams, and were asked to choose the appropriate item to fill in the missing part.

Faux Pas Test (FP) [47], which consists of 20 stories including 10 faux pas and 10 control stories, was used to assess the first and second order of theory of mind (ToM). During the test, a research assistant read 20 stories to the participants in random order, and then the participants were asked to answer three to six questions (FP Q1–Q6), depending on whether a faux pas was detected (Table 1). In short, FP Q1 measured the ability of the participants to detect a faux pas (detection); FP Q2 measured their ability to identify the person who committed the faux pas (identification); FP Q3 measured their ability to interpret the recipient’s mental state; FP Q4 measured their ability to understand the speaker’s intention; and FP Q5 and Q6 measured their ability to recall specific story content.

**Table 1:** Questions of faux pas test

	**Participants were asked to answer the following questions for each story read by the research assistant:**
Q1.	Detect a faux pas: “In the story, did anyone say something that they should not have said or something awkward?”
Q2.	Identify who committed the faux pas: “Who said something that they should not have said or something awkward?”^a^
Q3.	Interpret the recipient’s mental state: “Why shouldn’t he/she have said it?”^a^
Q4.	Understand the speaker’s intention: “Why do you think he/she said it?”^a^
Q5. & Q6.	Recall specific story content, for example: “Who won the competition?”^b^

^a^This question was only asked if the answer to Q1 was yes.

^b^Control questions.

The participants needed to answer questions FP Q2 to Q4 as listed above only if they detected a faux pas (FP Q1) in the stories (both faux pas and control stories). If no faux pas was detected, FP Q2 to Q4 were omitted. In either case, the control questions (FP Q5 and Q6) were administered to the participants. One point was given for each correct answer, and zero points were given otherwise. Four subscores (FP Q1–Q4) were computed for analysis in the present study by summing the points awarded in 10 faux pas stories, and the total score was derived by the summation of the four subscores (FP Q1–Q4 ). All scores (FP Q1–Q6) for the 10 control stories as well as the “control questions” (FP Q5–Q6) for the 10 faux pas stories were not used for analyses in the present study. In the present study, the reliabilities for this test were 0.74–0.76 and 0.71–0.83 for healthy controls and people with schizophrenia, respectively.

Reading the Mind in the Eyes Test (Eyes Test) [48] was administered to examine ToM or the participants’ automatic decoding abilities. In this task, participants were shown a total of 36 black-and-white eye pictures depicting various mental states. After each stimulus presentation, they were asked to choose from four choices the most appropriate mental state description (e.g., “upset”) for each eye picture. The reliabilities obtained were 0.17 (healthy controls) and 0.52 (people with schizophrenia). The low reliability of this test for the former group was expected due to the ceiling effect found in nonclinical participants [49].

The Interpersonal Reactivity Index (IRI) [50] was administered to measure empathy. This 28-item IRI consists of four subscales assessing both cognitive and emotional empathy. Only the total score, derived by summing all four subscores, was used for analysis in the present study. Its reliabilities in healthy controls (α = 0.73) and people with schizophrenia (α = 0.70) were good in the present study.

Positive and Negative Syndrome Scale (PANSS) [51] was administered to the clinical participants to assess symptom severity in adults with schizophrenia, schizoaffective disorder, or other psychotic illnesses. A total of 30 items consists of three subscales: positive, negative, and general psychopathology. The original three-factor PANSS was used for analysis in the present study, and the reliabilities were 0.56 (positive), 0.77 (negative), and 0.58 (general).

### Statistical analysis

Between-group differences (schizophrenia group vs. control group) in terms of neurocognitive and social-cognitive measures were tested with multivariate analysis of variance (MANOVA). Specifically, the dependent variables included for MANOVA were neurocognitive (RPM, JLOT, and TOL) and social-cognitive (FP subscores and IRI) measures. Also, age, sex, and education were included as covariates in all MANOVA and mediation analyses. In the schizophrenia group, mediation of social cognition in the relation between neurocognition and symptomatology was examined using SPSS PROCESS macro [52] with the bootstrapping method outlined by Shrout and Bolger [53]. SPSS PROCESS macro is a computational tool for path analysis-based moderation and mediation analysis as well as their combination as a “conditional process model.” Various measures of effect size for indirect effects are generated in mediation models, along with bootstrap confidence intervals for effect size inference [52]. Specifically, the SPSS PROCESS macro commands were used to compute the direct estimate of neurocognitive functioning on psychiatric symptoms (path (C) listed in Table 2) as well as the total (path (C) + (C’) listed in Table 2) and specific indirect effects (path (C’) listed in Table 2) of neurocognitive functioning on psychiatric symptoms through social cognitive functioning. Age, education, and sex were included in the model as covariates. However, duration of illness and onset of illness were not included as covariates in the mediation analyses because their correlations with all major variables including “perception,” “execution functioning,” “empathy,” “theory of mind,” and “symptomatology” (negative, positive, and general symptoms) were not significant (Table 3). Bootstrapping was used to estimate indirect effects, and 95% bias-corrected confidence intervals were used for the indirect effects using 1,000 bootstrap samples [53].

**Table 2:** Statistical tests for the social cognition mediation in neurocognition- symptomatology relation

**Neurocognitive functioning- social cognitive functioning**	**Psychiatric symptoms**	**A Estimate (P-value)**	**B Estimate (P-value)**	**C Estimate (P-value)**	**C’ Estimate (P-value)**	**Indirect effect (Lower limit 95% CI, upper limit 95% CI)**	**Effect size of the indirect effect**	***Controlling for education, age and sex***	**A Estimate (P-value)**	**B Estimate (P-value)**	**C Estimate (P-value)**	**C’ Estimate (P-value)**	**Indirect effect (Lower limit 95% CI, upper limit 95% CI)**
Perception- ToM	Negative symptoms	0.36** (0.0053)	-0.99* (0.0227)	-0.97* (0.0201)	-0.62 (0.1469)	-0.3553 (-0.9668, -0.0480)	0.0603		0.33* (0.0274)	-0.97* (0.0302)	-1.03* (0.0334)	-0.71 (0.1399)	-0.3159 (-1.0705, -0.0077)
Executive functioning - ToM		0.26* (0.0200)	-1.03* (0.0159)	-0.78* (0.0290)	-0.52 (0.1464)	-0.2667 (-0.6880, -0.0518)	0.0498		0.23* (0.0405)	-0.99* (0.0266)	-0.76* (0.0405)	-0.53 (0.1491)	-0.2279 (-0.6625, -0.0149)
Perception- ToM	Positive symptoms	0.36** (0.0053)	-0.62* (0.0520)	-0.14 (0.6448)	0.08 (0.7849)	-0.2199 (-0.6595, -0.0223)	0.0026		0.33* (0.0274)	-0.64* (0.0530)	-0.20 (0.5676)	0.01 (0.9804)	-0.2071 (-0.7150, 0.0044)
Executive functioning - ToM		0.26* (0.0200)	-0.49 (0.1097)	-0.39 (0.1158)	-0.27 (0.2991)	-0.1261 (-0.3579, -0.0118)	0.0254		0.23* (0.0405)	-0.54 (0.0947)	-0.42 (0.1129)	-0.29 (0.2743)	-0.1238 (-0.3888, -0.0027)
Perception – ToM	General symptoms	0.36** (0.0053)	-0.55 (0.1300)	-0.53 (0.1256)	-0.32 (0.3659)	-0.1978 (-0.6631, 0.0324)	0.0275		0.33* (0.0274)	-0.53 (0.1554)	-0.65 (0.1030)	-0.47 (0.2469)	-0.1735 (-0.7176, 0.0364)
Executive functioning - ToM		0.26* (0.0200)	-0.46 (0.1797)	-0.69* (0.0162)	-0.57* (0.0545)	-0.1204 (-0.3798, 0.0361)	0.0377		0.23* (0.0405)	-0.46 (0.2045)	-0.71* (0.0169)	-0.60* (0.0477)	-0.1061 (-0.4127, 0.0351)

Note: (A) Regression slope of neurocognitive functioning predicting social cognitive functioning; (B) regression slope of social cognitive functioning predicting psychiatric symptoms, controlling for neurocognitive functioning; (C) regression slope of neurocognitive functioning predicting psychiatric symptoms; (C’) regression slope of neurocognitive functioning predicting psychiatric symptoms, controlling for social cognitive functioning. Bootstrapping was used to estimate indirect effects (Shrout & Bolger, 2002).

*P < = 0.05.

**P < =0.01.

**Table 3:** Zero-order correlations among major variables and demographics in participants with schizophrenia

	**1**	**2**	**3**	**4**	**5**	**6**	**7**	**8**	**9**	**10**	**11**
1 Duration of illness	1	-	-	-	-	-	-	-	-	-	-
2 Age of onset	-0.43**	1	-	-	-	-	-	-	-	-	-
3 Age	0.55**	0.43**	1	-	-	-	-	-	-	-	-
4 Education	-0.05	-0.07	-0.17	1	-	-	-	-	-	-	-
5 Perception	0.14	-0.11	0.01	0.45**	1	-	-	-	-	-	-
6 Executive functioning	0.09	-0.03	-0.01	0.17	0.31*	1	-	-	-	-	-
7 Theory of mind	-0.07	-0.01	-0.13	0.27*	0.36**	0.31*	1	-	-	-	-
8 Empathy	0.05	-0.02	0.05	-0.13	-0.07	0.41**	0.02	1	-	-	-
9 Negative symptoms	-0.08	0.04	0.00	-0.17	-0.31*	-0.29*	-0.38**	-0.06	1	-	-
10 Positive symptoms	0.00	-0.07	-0.01	0.00	-0.06	-0.21	-0.26*	-0.13	0.14	1	-
11 General symptoms	-0.22	0.14	0.02	-0.08	-0.20	-0.31*	-0.26*	0.10	0.48**	0.58**	1

*P < = 0.05.

**P < = 0.01.

## Results

### Social and neurocognitive abilities across groups

One-way MANOVA results showed that healthy controls had significantly better neurocognitive (Wilk’s *λ* = 0.55, *F*_5, 110_ = 17.87, *P* < 0.001) and social cognitive (Wilk’s *λ* = 0.69, *F*_6, 109_ = 8.09, *P* = 0.000) abilities than the schizophrenia group had after controlling for age, sex, and education. In terms of neurocognitive abilities, healthy controls performed significantly better on the RPM, JLOT, and all three TOL measures (*Ps* < 0.05) after controlling for age, sex, and education. These were consistent with prior findings [2,3]. However, after adding the social cognition abilities (FP Q1–Q4 and Eyes Test scores) as covariates, the effect was not significant for all three TOL subscores (*P* > 0.05). For social cognitive abilities, healthy controls performed significantly better on the detection (FP Q1) and identification (FP Q2) of faux pas as well as on the Eyes Test (*Ps* < 0.05) after controlling for age, sex, education, and neurocognitive abilities (RPM, JLOT, and all three TOL subscores). These were consistent with the prior findings [26,27]. The group differences were not significant in terms of the ability to interpret a recipient’s mental states (FP Q3) and understand a speaker’s intention (FP Q4) or empathy measured by IRI (*Ps* > 0.05). These were not consistent with previous findings [26,28]. These between-group results are summarized in Table 4.

**Table 4:** Mean (SD) comparison of major variables after controlling for sex as covariates

	**Healthy controls (n = 61)**	**Schizophrenia (n = 58)**	**F-statistic/group difference *P*-values**	**Partial Eta Squared**	***Controlling for age, sex, education and neurocognitive/ social cognitive measures***	**F-statistic/group difference *P*-values**	**Partial Eta Squared**
*Neurocognitive measures*^*a*^			17.87***	0.45		9.33***	0.31
Raven’s PM	49.8 (7.3)	34.9 (10.6)	0.000***	0.41		0.000***	0.29
JLOT	24.1 (4.7)	19.0 (6.4)	0.000***	0.16		0.04*	0.04
TOL							
Total time (ms)^b^	492.2 (131.3)	563.5 (144.4)	0.003**	0.08		0.08^c^	0.03
Total score	16.7 (3.7)	14.2 (4.1)	0.002**	0.09		0.08^c^	0.03
Accuracy	1.91 (0.5)	1.7 (0.6)	0.34*	0.40		0.33^c^	0.01
*Social-cognitive measures*^*a*^			8.09***	0.31		2.69*	0.13
FP Q1 (detection)	7.4 (2.3)	5.8 (2.5)	0.001**	0.10		0.03*	0.04
FP Q2 (identification)	7.4 (2.4)	5.6 (2.5)	0.000***	0.12		0.02*	0.05
FP Q3 (others’ mental states)	2.8 (2.5)	3.3 (2.8)	0.34^c^	0.01		0.75^c^	0.001
FP Q4 (intention)	4.1 (2.7)	3.2 (2.4)	0.10^c^	0.02		0.79^c^	0.001
ET	22.7 (3.1)	19.1 (4.1)	0.000***	0.18		0.04*	0.04
Empathy	62.0 (11.4)	62.2 (12.2)	0.96^c^	0.00		1.00^c^	0.000

^a^Raven’s PM = Raven’s Progressive Matrices Test, JLOT = Judgment of Line Orientation Test, TOL = Tower of London Test, FP = Faux Pas, and ET = Eyes Test; ^b^ms = millisecond; ^c^n.s. = not significant.

*P < = 0.05; **P < = 0.01; ***P < = 0.001.

### Mediation analyses in people with schizophrenia

The present study adopted the well-established factor structure of neurocognition (perception and executive functioning) and social cognition (theory of mind (ToM) and empathy) for mediation analyses. For the mediation analyses, the composite scores of neurocognition and social cognition while the separate subscores of symptomatology (negative, positive, and general) were used. Specifically, the composite score of perception was computed by taking the sum of the standardized scores of RPM and JLOT, while the execution functioning composite score was calculated by summing up three standardized subscores of TOL with the score reversion of TOL total time. The composite score of ToM was computed by taking the sum of the standardized scores of FP and ET, while empathy was indexed by the total IRI score. Since the correlations of empathy with neurocognitive and social cognitive composite scores were not significant except for the association with the executive functioning composite score (*r* = .41, *P* < 0.01) (Table 3), the mediation analyses were only performed for the ToM composite score but not with empathy as a mediator. A confidence interval that does not contain zero indicates statistically significant mediation (*P* < 0.05) [53]. The mediation statistics are summarized in Table 2.

### Negative symptoms as DV

ToM fully mediated the relation between neurocognitive abilities (perception or executive functioning) and negative symptoms after controlling for all covariates (95% CI = -1.0705 to -0.0077 for perception; 95% CI = -0.6625 to -0.0149 for executive functioning) (Table 2). Perception (β = -1.03, *P* = 0.03) and executive functioning (β = -0.76, *P* = 0.04) as well as ToM (βs = -0.97, -0.99, *Ps* = 0.03) were negatively associated with negative symptoms, which was expected [2-4,26,54]. The association of perception (β = -0.71, *P* = 0.14) and executive functioning (β = -0.53, *P* = 0.15) with negative symptoms was abolished after controlling for ToM and all covariates.

### Positive symptoms

ToM fully mediated the relation between executive functioning and positive symptoms after controlling for all covariates (95% CI = -0.3888 to -0.0027). Executive functioning (β = -0.42, *P* = 0.11) and ToM (β = -0.54, *P* = 0.09) were negatively associated with positive symptoms, which was also expected [26,54]. However, such mediation was not significant for the perception–positive symptoms relation (95% CI = -0.7150 to 0.0044) (Table 2).

### General symptoms as DV

ToM did not significantly mediate the relation between neurocognitive abilities (perception or executive functioning) and general symptoms (95% CI = -0.7176 to 0.0364 for perception; 95% CI = -0.4127 to 0.0351 for executive functioning) with or without controlling for all covariates (Table 2).

## Discussion

To the best of our knowledge, this is the first study attempting to explore the role of social cognition in the relation between neurocognition and a broader spectrum of symptomatology in people with schizophrenia. Overall findings were consistent with prior studies’ findings that neurocognition and social cognition are impaired in schizophrenia [1,24]. More importantly, it was found that theory of mind (ToM) was a significant mediator explaining the relation of neurocognition to negative and positive symptoms [9]. However, empathy was not a significant mediator of such a relation, which is contrary to our hypothesis and to prior findings [22,37,38]. Overall findings suggest that social cognitive ability is one of the underlying factors of the neurocognition-symptomatology relation in schizophrenia, which has clinical implications for more cost-effective interventions for schizophrenia.

As expected, healthy controls had significantly better performance on perception, executive functioning [1], and all ToM measures [24], except for reasoning and inference of others’ mental states. These suggest that neurocognition, specifically the ability to perceive and understand the surrounding environment, along with visuo-spatial processing, planning, and problem-solving skills are impaired in people with schizophrenia. Furthermore, these findings suggest that these people also have social cognitive deficits in which they lack the ability to detect a faux pas and identify the person who has committed the faux pas in a social interaction.

However, empathy as measured by IRI was not impaired in these people, which is contrary to our hypothesis. One possible reason for the insignificant differences across groups might be that IRI [50] is not sensitive enough to detect such social cognitive deficits in schizophrenia in the Chinese context. Another reason might be that IRI is a self-report empathy scale, and schizophrenic subjects might be biased about their self-ratings on their social cognitive abilities. Thus, it might be more sensitive to detect the difference using objective empathy measures. Third, the inconsistent results might also be due to the fact that possible aspects such as age and education level of the participants were not accounted for in the group comparison analyses in prior findings [28] while the medication conditions were not accounted for in the present study. These speculations should be investigated in future studies.

In attempting to explain the relation between neurocognitive abilities and negative or positive symptoms in schizophrenia, ToM was found to be a full mediator. Specifically, the findings of the present study suggest that impairments in perception or executive functioning worsen the interpretation and understanding of others’ mental states or intentions. This in turn leads to more negative symptoms in schizophrenia [6,15]. In other words, the deficits in the organization, identification, and interpretation of sensory information in order to represent and understand the environment as well as problem-solving skills affect the ability to detect social cues and faux pas while interacting with others in schizophrenia. This in turn affects their social functioning including their ability to experience pleasure in their social interactions. In addition, it is suggested that impaired executive functioning leads to more positive symptoms through increased ToM deficits in schizophrenia. This suggests that the lack of ability to control and regulate cognitive processes such as working memory, reasoning, task flexibility, and execution in people with schizophrenia worsens their capacity to detect social cues and faux pas while interacting with others. This further leads to more abnormal outcomes such as hallucinations, delusions, and bizarre behaviors in these people.

Contrary to our expectation [35], ToM could not explain the relation between perception and positive symptoms because perception was not related to positive symptoms (r = -0.06) in the present study [11]. This inconsistent finding might be because the strength of the relation between positive symptoms and different neurocognitive domains varies or because different neurocognitive measures were used in prior studies [11,15,16]. On the other hand, theory of mind (ToM) did not mediate the relation between neurocognition and general psychopathological symptoms, which was not expected. This finding suggests that the ability to understand and identify others’ mental states and intentions does not explain the relation of neurocognition to general symptoms in schizophrenia. One of the reasons for this finding is that the general psychopathological symptoms encompass a variety of symptoms, and it is possible that some of these symptoms are more closely related to certain domains of neurocognition and social cognition but that others are not. For instance, poor insight might be more associated with ToM than with empathy. Future studies should further examine the relation of specific general symptoms with neurocognition and social cognition. Furthermore, another social cognitive domain, empathy, was not related to any psychiatric symptoms, which is also inconsistent with prior findings. This might be because this domain was evaluated by different measures in different psychotic disorders previously [22,37-39].

### Limitations

The current study suffers from five major limitations. First, the mediation analyses performed with neurocognition, social cognition, and symptomatology were based on cross-sectional data. Therefore, we could not conclude the causality of the mediated relationship found in the present study. However, the current study is an essential step toward confirming the association between neurocognition, social cognition, and symptomatology in schizophrenia before a longitudinal study in perception is undertaken. Second, due to the time constraints for assessing each participant, empathy and executive functioning were each only assessed by one scale, the Interpersonal Reactivity Index [50] and the Tower of London [43], respectively. This might not fully capture the whole dimension of empathy and executive functioning. Third, the present study only included theory of mind and empathy as the concept of social cognition, while only perception and executive functioning were included to represent neurocognition (also because of the time constraint factor). Future studies should include more measurement tools so to fully represent these concepts. For instance, social knowledge and attribution styles, which are social-cognitive domains and often neglected in prior studies, warrant further investigation [9]. Also, working-memory and attention processes should also be assessed in the future. Fourth, the self-report measure was used to assess empathy in the present study while the other social cognition domain, theory of mind, was tested by objective measures. Since there might be bias in self-ratings of empathy by schizophrenia [55]*,* further studies should address this limitation in the future. Last but not least, the classic chlorpromazine (CPZ) equivalents that can be used to chart relative antipsychotic potencies of antipsychotic drugs were not recorded in the present study. Since it might be a factor in symptomology in schizophrenia, future studies should also include CPZ equivalents as a covariate.

## Conclusions

By examining a broader concept of social cognition and symptomatology and by separating neurocognition into various factors, we could identify specific social and neurocognitive impairment in schizophrenia. More importantly, the findings suggest that neurocognitive impairment predisposes people with schizophrenia to deficits in theory of mind, which in turn increases symptomatology, including negative and positive symptoms in schizophrenia. In other words, we could better enhance clinical outcomes by improving ToM ability in therapeutic treatment pertaining to people with schizophrenia. For instance, an integrated therapeutic approach with the combination of neurocognition and social cognition [56,57] might be a more effective approach to treating the symptomatology of people with schizophrenia.

## Competing interests

All authors report no conflicts of interest relevant to the subject of this article.

## Authors’ contributions

TMCL conceptualized the research idea. All authors designed the study. TMCL collected the clinical data. BYHL analyzed the data and wrote the first draft of the manuscript. TMC and AR supervised the research project and critically revised the manuscript. All authors contributed to and have approved the final manuscript.

## Pre-publication history

The pre-publication history for this paper can be accessed here:

http://www.biomedcentral.com/1471-244X/14/138/prepub
